# The weight, urine colour and thirst Venn diagram is an accurate tool compared with urinary and blood markers for hydration assessment at morning and afternoon timepoints in euhydrated and free-living individuals

**DOI:** 10.1017/S000711452300274X

**Published:** 2024-04-14

**Authors:** Marcos S. Keefe, Hui-Ying Luk, Jan-Joseph S. Rolloque, Nigel C. Jiwan, Tyler B. McCollum, Yasuki Sekiguchi

**Affiliations:** 1 Sports Performance Laboratory, Department of Kinesiology and Sport Management, Texas Tech University, Lubbock, TX 79407, USA; 2 Applied Physiology Laboratory, Department of Kinesiology and Sport Management, Texas Tech University, Lubbock, TX, USA

**Keywords:** Dehydration, Hydration assessment, Field settings

## Abstract

The weight, urine colour and thirst (WUT) Venn diagram is a practical hydration assessment tool; however, it has only been investigated during first-morning. This study investigated accuracy of the WUT Venn diagram at morning and afternoon timepoints compared with blood and urine markers. Twelve men (21 ± 2 years; 81·0 ± 15·9 kg) and twelve women (22 ± 3 years; 68·8 ± 15·2 kg) completed the study. Body mass, urine colour, urine specific gravity (USG), urine osmolality (U_OSM_), thirst and plasma osmolality (P_OSM_) were collected at first-morning and afternoon for 3 consecutive days in free-living (FL) and euhydrated states. Number of markers indicating dehydration levels were categorised into either 3, 2, 1 or 0 WUT markers. Receiver operating characteristics analysis calculated the sensitivity and specificity of 1, 2 or 3 hydration markers in detecting dehydration or euhydration. Specificity values across morning and afternoon exhibited high diagnostic accuracy for USG (0·890–1·000), U_OSM_ (0·869–1·000) and P_OSM_ (0·787–0·990) when 2 and 3 WUT markers were met. Sensitivity values across both timepoints exhibited high diagnostic accuracy for USG (0·826–0·941) and U_OSM_ (0·826–0·941), but not P_OSM_ in the afternoon (0·324) when 0 and 1 WUT markers were met. The WUT Venn diagram is accurate in detecting dehydration for WUT2 and WUT3 based off USG, U_OSM_ and P_OSM_ during first-morning and afternoon. Applied medical, sport and occupational practitioners can use this tool in field settings for hydration assessment not only at various timepoints throughout the day but also in FL individuals.

Adequate hydration status, widely termed ‘euhydration’, can be defined as maintaining a normal total body water balance^(1)^. In contrast, dehydration is essentially a result of total body water deficit, which may lead to negative physiological and exercise performance^(2)^. Maintenance of euhydrated (EUH) status alludes to the importance of proper and efficient assessment in various scenarios, including sport, occupational and military settings. While several methodological assessments exist to measure hydration status, there is currently no consensus amongst the scientific community regarding an unambiguous gold standard method, notably regarding implementation in field settings^(3)^.

Armstrong (2005 & 2007) has extensively reviewed commonly practiced hydration assessment techniques for laboratory and field settings, mainly involving the use of whole-body, haematologic, urinary and sensory indices^(4,5)^. These measures involve varying levels of technique complexity and differ greatly in applicability because of measurement reliability, equipment cost, accuracy of detecting meaningful hydration status fluctuations and the type of anticipated dehydration experienced^(3,6,7)^. For field settings where it is impractical to use invasive measures or laboratory equipment, a summative model involving three feasible variables was proposed for practical hydration assessment.

A Venn diagram decision tool that combines three of the simplest hydration markers, consisting of weight, U_COL_ and thirst (WUT), was first proposed by Cheuvront and Sawka (2005) as an applicable and cost-efficient strategy to assess hydration status in athletes^(3)^. This model was designed based on scientific principles of these three markers correlating with other urinary and blood indices for hydration assessment^(3)^. No marker individually provides sufficient evidence of predicted dehydration, but the combination of any two markers may indicate an individual is ‘likely dehydrated’^(1,3,8)^. When all three markers are met, then an individual is ‘very likely dehydrated’^(1,3,8)^. Sekiguchi *et al.* (2022) validated the WUT Venn diagram in relationship to urinary hydration indices (urine specific gravity (USG) and U_OSM_); however, this tool has not been validated with haematologic indices, which may provide greater utility for clinical hydration assessment in athletes compared with urinary markers^(2,9)^. In addition, the relationship between the WUT Venn diagram and other hydration markers has only been assessed using first-morning measurements. Although literature suggests that first-morning samples should be used to establish baseline measurements for body mass^(10)^, some researchers suggest that a first-morning urine spot sample should not be used for hydration assessment to detect hydration status throughout the day^(11)^. In contrast, Bottin *et al.* (2016) demonstrated that mid- to late-afternoon urine spot samples produced equivalent values to 24-h urinary indices^(12)^. Often, athletes or military personnel are unable to assess first-morning samples due to confounding factors (e.g. travelling, early-morning meetings/practices). Additionally, these populations frequently exercise in the afternoon, but a first-morning spot sample likely does not reflect hydration status at the afternoon timepoint. Thus, the potential use of the WUT Venn diagram at an afternoon timepoint creates more flexibility and practicality for hydration assessment in field settings.

Hydration assessment research generally has been conducted in EUH individuals to ensure reliability of techniques. However, athletes, military personnel or occupational workers are not always EUH in a real-life setting, leading to the need to investigate the Venn diagram’s applicability in free-living (FL) individuals as well. Therefore, the purpose of this study was to investigate the accuracy of the WUT Venn diagram at morning and afternoon timepoints compared with haematologic and urinary indices during EUH and FL conditions. We hypothesised that both morning and afternoon spot samples would demonstrate high accuracy when using the WUT Venn diagram to assess dehydration when two or three WUT variables are met.

## Experimental methods

### Ethical approval

This study was conducted according to the guidelines laid down in the Declaration of Helsinki, and all procedures involving human subjects/patients were approved by the Texas Tech University Institutional Review Board; 2022-640 (remove for review). Written informed consent was obtained from all subjects/patients.

### Participants

A total of twenty-four participants, twelve men (mean ± sd; age: 21 ± 2 years; mass: 81·0 ± 15·9 kg) and twelve women (age: 22 ± 3 years; mass: 68·8 ± 15·2 kg), volunteered to participate in this study. All participants reported not having kidney disease or a urinary tract infection at the time of the study. Only women using an oral contraceptive pill were recruited to participate in this study and completed the study visits during the 7-d placebo pill time frame. This was designed to ensure all participation from female participants was completed during the menstruation phase of the menstrual cycle.

### Procedures

Participants visited the laboratory twelve occasions across a 7-d time frame. Visits were performed in the morning and afternoon of three consecutive days in a FL situation. Researchers instructed participants to maintain their habitual lifestyle during the FL condition, including eating, drinking and exercising. Following a 1-d break, the remaining visits were performed in the morning and afternoon for three consecutive days in a EUH state. Euhydration was defined as providing a spot urine sample with a USG < 1·020. Researchers provided participants with fluid intake reminders throughout the days every 3 h to ensure participants would be EUH during these visits. Table 1 demonstrates hydration state according to the hydration variables during the FL and EUH conditions. This study design order of completion (i.e. first 3 d FL; second 3 d EUH) was implemented to minimise potential crossover effects of enforced euhydration during the EUH visits. In addition, irrespective of condition state (FL or EUH), participants were instructed to complete fluid and food logs between the morning and afternoon visits.

**Table 1.** Hydration marker (body mass (BM), BM loss (BML), urine colour (U_COL_), urine specific gravity (USG), urine osmolality (U_OSM_) and plasma osmolality (P_OSM_)) descriptive values at morning and afternoon timepoints for both euhydrated (EUH) and free-living (FL) conditions

	EUH morning		EUH afternoon		FL morning		FL afternoon	
	Mean	sd	Mean	sd	Mean	sd	Mean	sd
BM (kg)	75·03	16·10	75·37	16·17	74·91	16·08	75·13	16·00
BML (%)	0·0	0·5	−0·5	0·8	0·1	1·0	−0·2	1·1
U_COL_	3	1	2	1	5	2	4	2
Thirst	4	2	2	1	5	2	3	2
USG	1·011	0·004	1·008	0·006	1·019	0·008	1·014	0·008
U_OSM_ (mOsm)	406	158	334	207	690	276	589	293
P_OSM_ (mOsm)	287	5	286	6	287	5	286	6

All morning visits were performed as a first-morning spot sample. Participants were instructed to arrive to the laboratory abstaining from any food or fluid consumption and not to perform exercise. Urine cups were provided to participants the day prior so that the first-morning urine sample could be provided to researchers upon visitation to the laboratory. U_COL_, USG and U_OSM_ were then measured from the first-morning spot sample. Participants were then shown a Likert-type scale and were asked their current thirst sensation based off the scale. Following this, body mass (BM) measurements were attained. Lastly, a single-stick blood sample via venipuncture into a 2 ml lithium-heparin tube (BD, Franklin Lakes, NJ, USA) was collected to measure P_OSM_. The blood sample was immediately centrifuged for 20 min at 3000 rpm at 9°C to separate plasma from red blood cells.

**Fig. 1.**
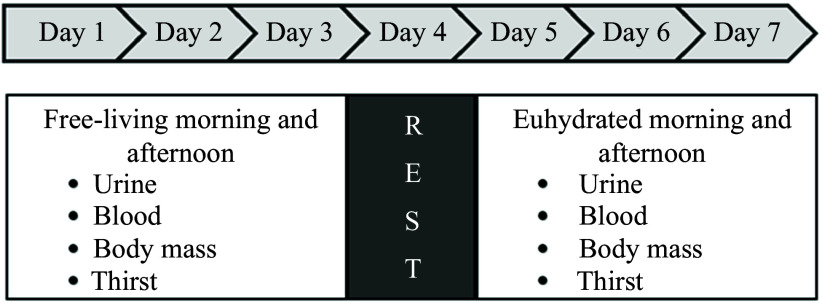
Experimental design timeline. Participants visited the laboratory in the morning and afternoon in a free-living condition for the first 3 d (Days 1–3). Following a 1-d break (Day 4), participants performed the remaining visits (Days 5–7) in a euhydrated condition (defined by a urine spot sample of USG < 1·020). Each visit consisted of attainment of a urine spot sample, blood sample, nude body mass measurement and thirst level.

All afternoon visits were performed using a spot sample between 2:00 and 4:00 pm and followed the exact procedures and design as the morning visits. However, participants were not fasted from fluid and food or abstained from exercise prior to the afternoon visits. Although the researchers did not control for food or fluid consumption prior to the afternoon visit, periodic reminders were delivered to participants to drink water to ensure euhydration during the three EUH visit days. Broad overview of the experimental design is illustrated in Fig. 1.

### Measurements

Urine indices (USG, U_OSM_ and U_COL_), haematologic indices (P_OSM_), body mass and thirst level were collected at each visit. USG was measured using a handheld refractometer (ATAGO, Tokyo, Japan), and U_COL_ was assessed via a validated eight-point U_COL_ chart^(13)^. U_OSM_ and P_OSM_ were analysed via an Advanced Instruments Osmometer Pro (Norwood, MA, USA), with each sample being measured in duplicate.

Nude BM was measured via an electronic scale (Health-o-Meter). Percentage BML for each day was calculated based on the average of the 3 EUH morning BM measurements for each participant: ((BM of each day – Baseline BM) × Baseline BM^-1^ × 100). Thirst level was assessed on a Likert-type scale of 1 to 9, with 1 being ‘not thirsty at all’ and 9 being ‘very, very thirsty’^(14)^.

### Weight, urine colour and thirst criteria determination

Dehydration thresholds were previously determined for the three WUT markers and if any of these criterions were met, then a score of ‘1’ was aggregated towards the final count. The total number of markers that indicated dehydration were counted and categorised into either 0, 1, 2 or 3 WUT markers (WUT0, WUT1, WUT2 and WUT3) and were compared with haematologic and urinary indices. A BML > 1 %, U_COL_ ≥ 5 and thirst level ≥ 5 were the designated dehydration thresholds^(1,15)^. In comparison with haematologic and urinary hydration markers, a USG ≥ 1·020, U_OSM_ > 700 mOsmol and P_OSM_ > 290 mOsmol indicate dehydration based on standards of the American College of Sports Medicine^(2)^.

### Statistical analyses and justification of sample size

A power analysis conducted with G * Power 3.1.9.7 (Universitat Kiel) determined that twenty-four participants were needed in the present study for a power of 0·80, with an effect size of 0·2 and an *α* level of 0·05^(15)^. Data are presented as mean ± se. Receiver operating characteristics analysis (i.e. sensitivity and specificity) was performed to calculate the predictive value of 1 (combined with 0), 2 or 3 hydration markers in detecting a dehydrated or EUH state, which were defined by USG, U_OSM_ and P_OSM_. Cut-off determination values were calculated based off the calculated sensitivity and specificity values^(16)^. Positive and negative predictive values provide additional context for the WUT indices to accurately predict hydration state according to USG, U_OSM_ and P_OSM_. High sensitivity corresponds to the WUT Venn diagram being accurate in determining euhydration, whereas high specificity corresponds with it accurately determining dehydration.

## Results

**Fig. 2.**
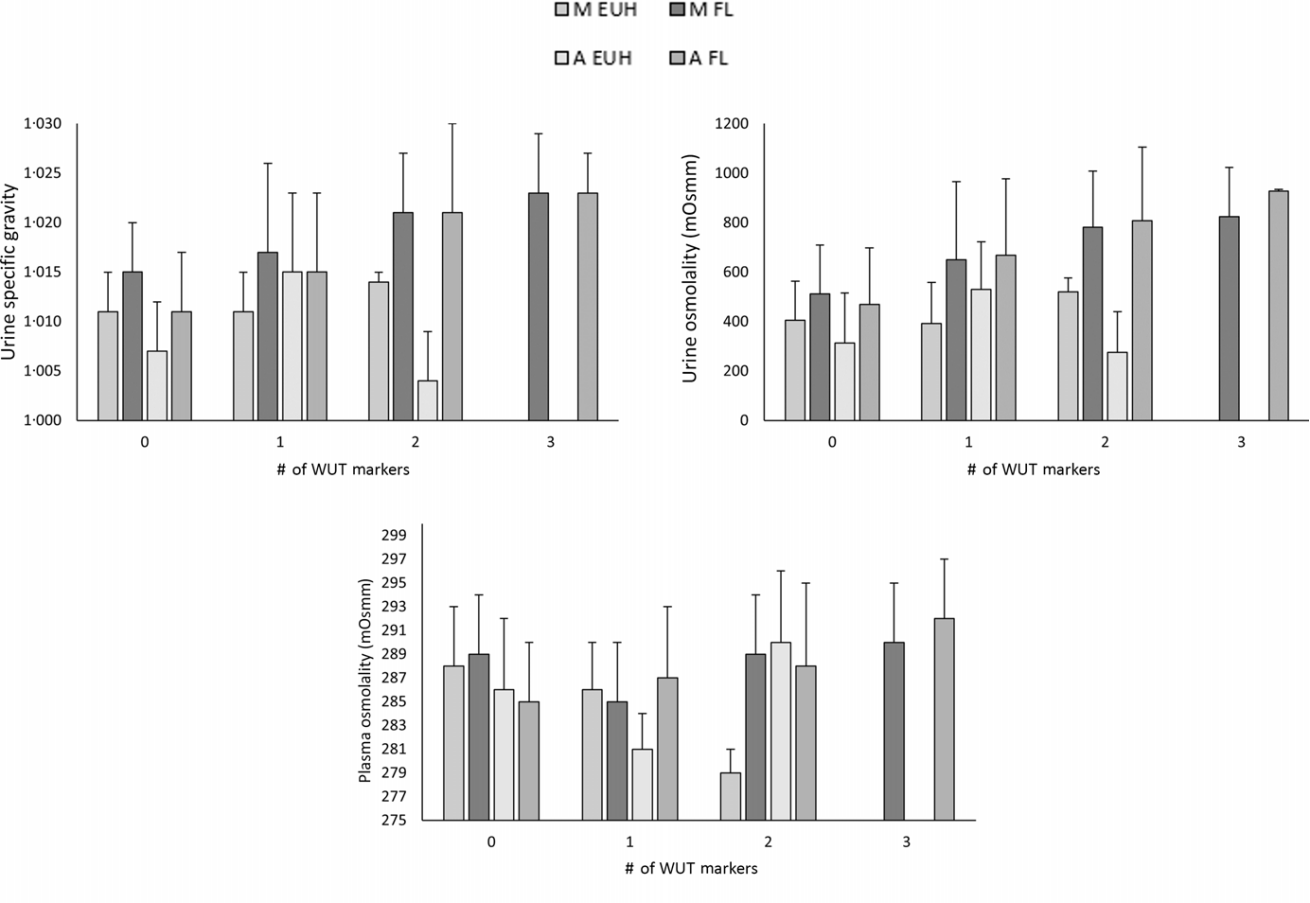
Morning (M) and afternoon (A) urine specific gravity, urine osmolality and plasma osmolality when weight, urine colour and thirst (WUT) Venn diagram criteria were used to determine hydration status. Data groups are split into morning and afternoon experimental conditions of free-living (FL) and euhydrated (EUH).

A total of 288 samples were analysed for USG and U_OSM_ and 271 samples for P_OSM_. Seventeen plasma samples were missed because of technical issues. Of the seventeen missed samples, six samples were missed during the morning visits of the EUH condition, four of afternoon EUH, four of morning FL and three of afternoon FL. A number of WUT markers met are shown in Table 2 between timepoints and conditions. Mean values of USG, U_OSM_ and P_OSM_ for each WUT category at each timepoint and condition are presented in Fig. 2.

**Table 2.** Categorisation of the number of samples in each weight, urine colour and thirst (WUT) category at morning and afternoon timepoints

Condition	Timepoint	WUT0	WUT1	WUT2	WUT3
N/A	Morning	50	59	29	6
	Afternoon	99	34	9	2
EUH	Morning	39	29	4	0
	Afternoon	63	7	2	0
FL	Morning	11	30	25	6
	Afternoon	36	27	7	2

### Receiver operating characteristics for morning and afternoon timepoints

**Fig. 3.**
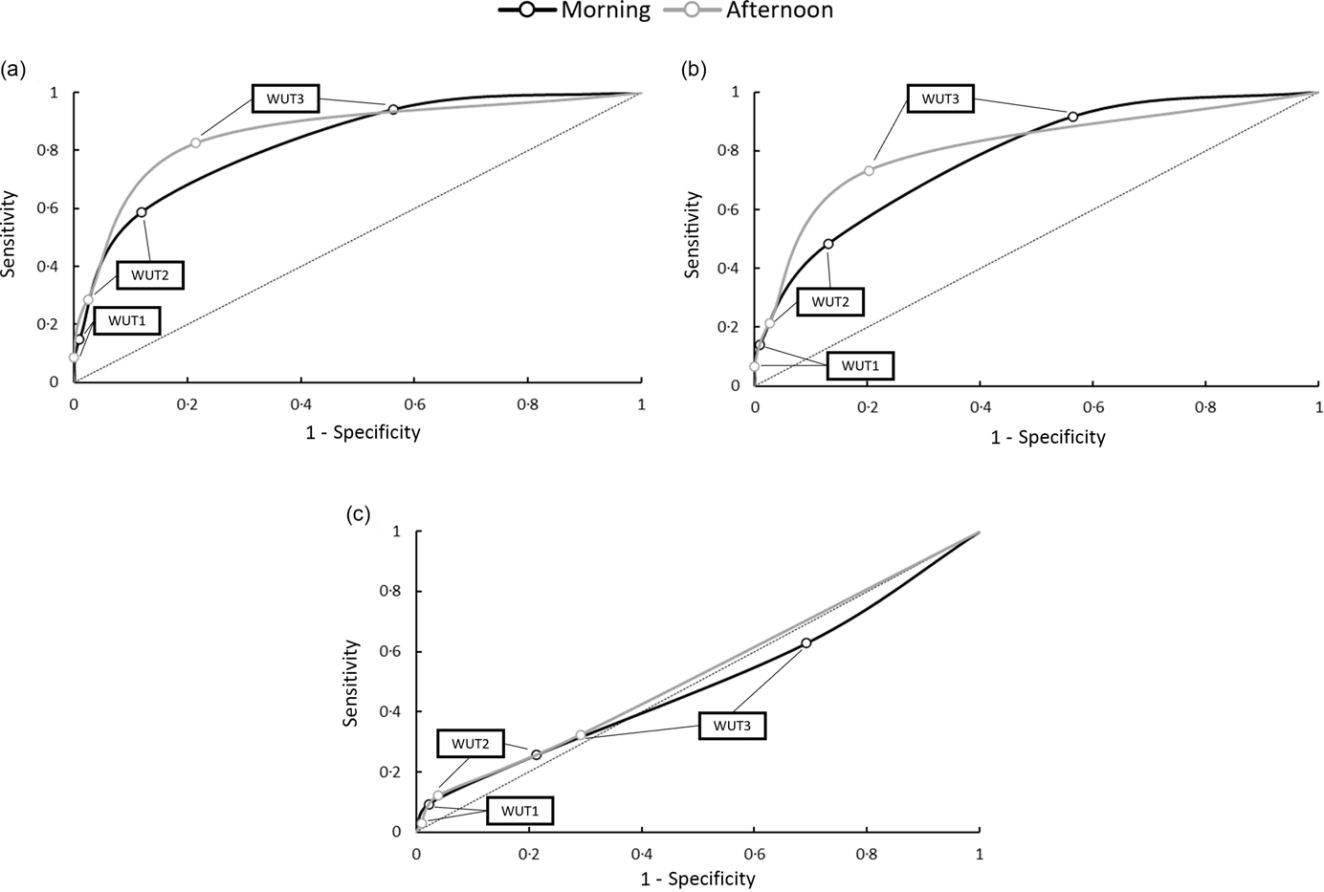
Receiver operating characteristic (ROC) curves from morning and afternoon timepoints for (a) urine specific gravity, (b) urine osmolality and (c) plasma osmolality. WUT1, WUT2 and WUT3 thresholds are plotted appropriately.

Fig. 3 presents sensitivity and specificity values in receiver operating characteristics figures for morning and afternoon timepoints when WUT criteria were used to determine hydration status in comparison to urinary and haematologic hydration variables (USG, U_OSM_ and P_OSM_). At both timepoints, WUT2 and WUT3 resulted in high specificity values in comparison to USG, U_OSM_ and P_OSM_. In addition, WUT1 resulted in high sensitivity values in comparison to USG and U_OSM_, but interestingly did not result in high sensitivity values for P_OSM_ at the afternoon timepoint.

**Fig. 4.**
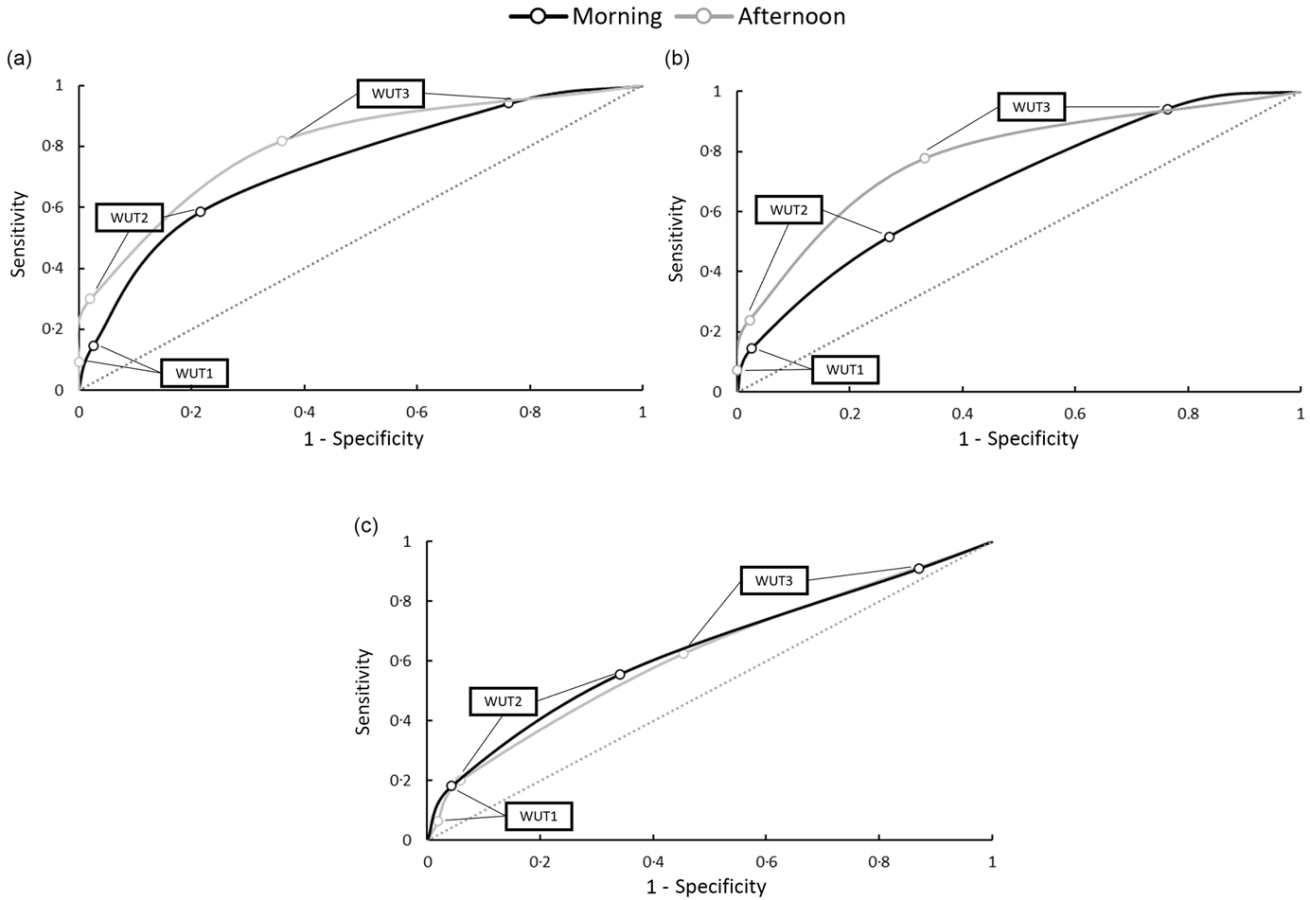
Receiver operating characteristic (ROC) curves from morning and afternoon timepoints for the free-living (FL) condition (a) urine specific gravity, (b) urine osmolality and (c) plasma osmolality. WUT1, WUT2 and WUT3 thresholds are plotted appropriately.

Fig. 4 presents sensitivity and specificity values in receiver operating characteristics figures for morning and afternoon timepoints when investigating the FL condition. Table 3 presents sensitivity and specificity values for morning and afternoon timepoints when investigating the EUH condition. At both morning and afternoon timepoints, WUT2 and WUT3 resulted in high specificity values in comparison to USG, U_OSM_ and P_OSM_ for both EUH and FL conditions. WUT1 resulted in high sensitivity values at both timepoints for the FL condition in comparison to USG, U_OSM_ and P_OSM_. However, WUT1 in the EUH condition did not result in a high sensitivity at either timepoint for P_OSM_, but did for U_OSM_, and was not applicable for USG.

**Table 3.** Morning and afternoon sensitivity, specificity, cut-off determination value, positive predictive value (PPV) and negative predictive value (NPV) for the euhydrated (EUH) condition’s urine specific gravity (USG), urine osmolality (U_OSM_) and plasma osmolality (P_OSM_) when weight, urine colour and thirst (WUT) Venn diagram criteria were used to determine hydration status (USG > 1·020, U_OSM_ > 700 mOsm and P_OSM_ > 290 mOsm)

	# of WUT markers	Sensitivity	Specificity	Cut-off determination value	PPV (%)	NPV (%)
Morning:Afternoon						
USG	3	-: -	1·000*: 1·000*	0·000:0·000	-: -	100·0:100·0
	2	-: -	0·944*: 0·972*	0·003:0·001	-: -	100·0:100·0
	1	-: -	0·542:0·875*	0·210:0·016	-: -	100·0:100·0
U_OSM_	3	-: -	1·000*: 1·000*	0·000:0·000	-: -	97·2:95·8
	2	-: -	0·943*: 0·971*	0·003:0·001	0:0	97·1:95·7
	1	0·500:0·333	0·543:0·884*	0·459:0·458	3·0:11·1	97·4:96·8
P_OSM_	3	-: -	1·000*: 1·000*	0·000:0·000	-: -	68·2:73·5
	2	-: 0·056	0·911*: 0·980*	0·008:0·892	0:50·0	66·1:74·2
	1	0·333:0·056	0·489:0·880	0·706:0·906	23·3:14·3	61·1:72·1

* Indicates sensitivity/specificity > cut-off determination value and > 0·800, which determines euhydration *v*. dehydration.

## Discussion

**Fig. 5.**
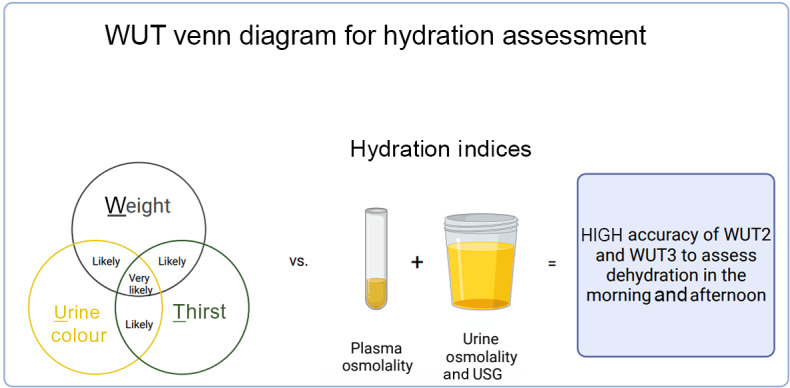
Weight, urine colour and thirst (WUT) Venn diagram is accurate for detecting dehydration at both morning and afternoon timepoints when two (WUT2) or three (WUT3) WUT variables are met in comparison to hydration indices of plasma osmolality, urine osmolality and urine specific gravity (USG). Created with BioRender.com.

This investigation examined the accuracy of the WUT Venn diagram in comparison to haematologic and urinary hydration indices at morning and afternoon timepoints. Findings of the present study support our hypothesis that an afternoon spot sample would result in high accuracy of the WUT Venn diagram in assessment of hydration status (Fig. 5). Results demonstrate that USG, U_OSM_ and P_OSM_ indices correspond with high specificity values at both morning and afternoon timepoints, indicating that the WUT Venn diagram is a valid indicator of dehydration for WUT2 and WUT3. Furthermore, these findings remained consistent when the EUH and FL condition data points were separated, still showing high specificity values for the three hydration indices to indicate dehydration for WUT2 and WUT3. To our knowledge, this is the first study to investigate accuracy of the WUT Venn diagram at an afternoon timepoint, and in both EUH and FL conditions.

Accuracy of the WUT Venn diagram has previously been established using a morning spot sample, but these studies solely compared the WUT variables to urinary indices^(15,17)^. Although plasma osmolality is not the gold standard variable for hydration assessment, haematologic indices may provide greater utility for clinical hydration assessment in athletes compared with other indices^(2,9)^. The current study expands upon previous literature demonstrating that meeting three WUT criteria is accurate in determining dehydration from high specificity values not only in comparison to urinary indices but also P_OSM_ at both morning and afternoon timepoints. Similarly, Wardenaar *et al.* (2023) confirmed that meeting all three WUT markers suggested a USG above the 1·020 cut-off and Sekiguchi *et al.* (2022) confirmed compared with USG and U_OSM_^(15,17)^. In addition, the current study demonstrates that meeting two WUT criteria is an accurate indication of dehydration. This is in contrast with previous findings, whereas Sekiguchi *et al.* (2022) stated that meeting only two WUT criteria may not be accurate in distinguishing dehydration from euhydration, especially when the two WUT criteria met were BML and thirst^(15)^. A potential explanation for these discrepancies is the separation of EUH and FL conditions examined in the current study. Altogether the morning and afternoon timepoints each demonstrated high accuracy of WUT2 for all three hydration indices, but these findings were further strengthened by both EUH and FL conditions also resulting in the same outcome. Sekiguchi *et al.* (2022) examined the accuracy of WUT2 with both hypohydrated and EUH data points combined, leading to potential overlap with one condition outweighing the other. Overall, these findings suggest that the WUT Venn diagram is an accurate tool to determine dehydration when 2 or 3 WUT criteria are met at various timepoints throughout the day. These results expand the application of this tool in real-world settings through demonstration of accuracy with an afternoon timepoint. Indeed, many athletic events/games regularly occur in the afternoon or evenings, thus it is important to accurately determine hydrations status in a feasible manner nearer to game times to ensure proper hydration.

The WUT Venn diagram was not initially designed as a tool to assess euhydration; however, we investigated this relationship by analysing WUT1. With both EUH and FL conditions combined, meeting one WUT criteria resulted in high sensitivity values for all three hydration indices in the morning, but only for USG and U_OSM_, not P_OSM_, in the afternoon. Furthermore, analysation of EUH and FL conditions separately demonstrated that WUT1 is not accurate in detecting euhydration at either timepoint during the EUH condition. In contrast, the FL condition demonstrated high sensitivity values at both timepoints when meeting one WUT criteria. The combination of these various findings suggests that interpretation should be carefully warranted if seeking to use the WUT Venn diagram as a tool for euhydration assessment. There remains a lack of literature regarding direct investigation of the WUT Venn diagram but results of the current study align with previous literature and conceptions that the combination of body mass and a urine concentration measurement allows ample accuracy for detecting dehydration when two or three WUT criteria are met^(8,15,17)^. Most hydration assessment research has been conducted in EUH individuals to ensure the reliability of such techniques. However, athletes, military personnel or occupational workers will not always be EUH in a real-life setting, leading to the design of this study to investigate the Venn diagram’s applicability in FL individuals as well.

During the FL condition, participants in this study were unlikely experiencing significant total body water losses or fluid shifts (> 2 % BML), whereas athletes (i.e. endurance athletes), military soldiers or occupational workers (i.e. construction workers, firefighters) may experience these in field settings, especially while in the heat or during exercise^(18)^. Thus, it is important to ensure that hydration assessment variables are also able to detect dehydration in these populations who would utilise a hydration assessment tool, such as the WUT Venn diagram. Although our study did not investigate these populations directly, a previous study showed that USG and UOSM identified 27–55 % of collegiate athletes as dehydrated, whereas a blood marker of serum Na concentration identified no athlete as dehydrated, attributing the lack of significant relationships between urine and blood markers to confounding effects of diet, timing of fluid intake and renal responses to exercise^(9,11)^. Indeed, a large and rapid intake of water acutely alters urinary indices, which may not be representative of an individual’s actual hydration state changing^(19)^. However, serum Na concentration is more so used as a clinical hydration marker of intracellular dehydration during extreme cases that require emergent treatment^(9,20–23)^, which may explain why Hew-Butler *et al*. (2018) did not classify any athletes as dehydrated through this marker^(9)^. According to the tool’s diagnostic thresholds, a BML (W aspect of the Venn diagram) greater than 1 % counts as 1 marker met, therefore individuals experiencing significant losses (>2 %) would still be meeting this marker. Additionally, to meet this significant level of BML, individuals would likely be exercising in the heat or performing a high-intensity bout of exercise^(18)^, where thirst and U_COL_ would also be likely increasing^(18,24)^ and meeting the WUT markers to signify dehydration.

There are a few potential limitations present in this study which may have influenced the findings. First, the WUT Venn diagram was tested in comparison to USG and U_OSM_, two variables that are derived from a urine sample. One of the Venn diagram’s three components is U_COL_, thus providing a direct linearity between a component of the WUT Venn diagram and two of the hydration markers we compared the tool to. P_OSM_ was included as a third external hydration marker to counter this limitation, thus findings of the present study may be stronger when assessing the relationship of the Venn diagram to P_OSM_, rather than USG and U_OSM_. In addition, BM was not controlled for in the afternoon setting. Due to the consumption of fluids and food, BM is likely to be higher in the afternoon, but this does not necessarily correlate with improved hydration status, as defined by an improved BML. We chose to not control for this as one of the research questions looked to investigate the accuracy of the Venn diagram in a FL condition, thus limiting controlling aspects by the researchers. However, the researchers acknowledge that BM may be a WUT variable that is not truly accurate in an afternoon setting. Lastly, although researchers controlled for the menstrual cycle, the inclusion of only female participants who were taking oral contraceptive pills and holding their visits during the placebo-pill week is a limitation for adapting these findings to females. This results in not having an inclusive adaptation of findings to females across the entire menstrual cycle, which is imperative to understand and where current literature is lacking.

In conclusion, the WUT Venn diagram is a practical hydration assessment tool that can assess dehydration by WUT2 and WUT3 in both the morning and afternoon. Although WUT1 may detect euhydration, discrepancies amongst condition type and urinary or haematologic hydration variables exist, thus limiting the strength of the Venn diagram tool for this type of assessment. In addition, results from the present study demonstrate that the Venn diagram is accurate in detecting dehydration in both EUH and FL individuals, increasing its applicability among different populations (e.g. athletes, military personnel and occupational workers). This tool ultimately provides a practical and cost-efficient strategy to accurately measure hydration status at various timepoints throughout the day.
